# COVID-19 confirmed patients with negative antibodies results

**DOI:** 10.1186/s12879-020-05419-3

**Published:** 2020-09-22

**Authors:** Jian Wang, Chong Chen, Qilin Li, Pengcheng Cai, Zheng Wang, Lin Wang

**Affiliations:** 1 Department of Clinical Laboratory, Union Hospital, Tongji Medical College, Huazhong University of Science and Technology, Wuhan, China; 2 Research Center for Tissue Engineering and Regenerative Medicine, Union Hospital, Tongji Medical College, Huazhong University of Science and Technology, Wuhan, China; 3 Department of Radiology, Union Hospital, Tongji Medical College, Huazhong University of Science and Technology, Wuhan, China; 4 Department of Gastrointestinal Surgery, Union Hospital, Tongji Medical College, Huazhong University of Science and Technology, Wuhan, China

**Keywords:** Case report, COVID-19, IgM, IgG, Negative antibodies results

## Abstract

**Background:**

A new coronavirus disease 2019 (COVID-19) has escalated to a pandemic since its first outbreak in Wuhan, China. A small proportion of patients may have difficulty in generating IgM or IgG antibodies against SARS-CoV-2, and little attention has been paid to them.

**Case presentations:**

We present two cases of confirmed COVID-19 patients and characterize their initial symptoms, chest CT results, medication, and laboratory test results in detail (including RT-PCR, IgM/ IgG, cytokine and blood cell counts).

**Conclusion:**

Both of patients with confirmed COVID-19 pneumonia failed to produce either IgM or IgG even 40 to 50 days after their symptoms onset. This work provides evidence demonstrating that at least a small proportion of patients may have difficulty in rapidly gaining immunity against SARS-CoV-2.

## Background

During the outbreak of coronavirus 2019 (COVID-19) [1–3], a small proportion of confirmed COVID-19 patients fail to produce IgM or IgG antibodies against SARS-CoV-2 even 40 days or longer periods of time after onset of their initial symptoms. However, most of the current studies so far are focused on the general population but for these patients.

From January 30 to March 15, 310 of COVID-19 patients who were positive for SARS-CoV-2 real time reverse-transcription PCR (RT-PCR) testing and received IgM and IgG detection at Wuhan Union Hospital (Wuhan, China) were enrolled. RT-PCR was performed through amplifying ORF1ab gene and N gene of SARS-CoV-2 (BioGerm, Shanghai, China) using oropharyngeal  swab specimens of all patients. From March 4 to 15, IgM and IgG of SARS-CoV-2 were tested using blood samples for all these 310 patients. Two different kits were used to detect antibodies through immune colloidal gold (ICG) technique (Yingnuote, Tangshan, China) and chemiluminescence immunoassay (CLIA) technique (Yahuilong, Shenzhen, China). Laboratory test results were collected and analyzed.

Among 310 COVID-19-confirmed patients, 308 of them were tested positive for IgM and/ or IgG, but only two patients were negative for IgM and IgG detection.

## Case presentations

### Case 1

**Fig. 1:**
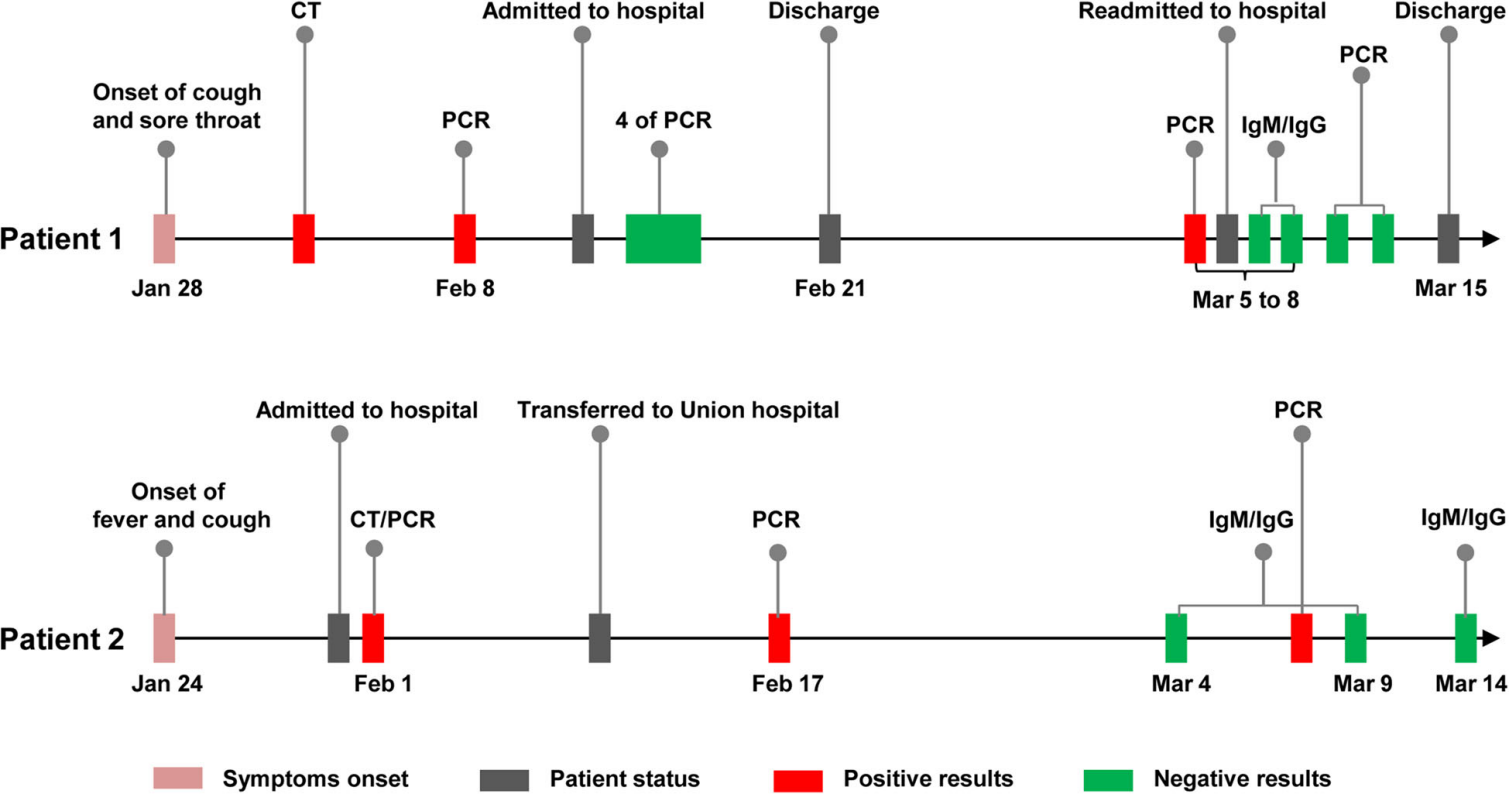
Chronology of symptom onset, hospital admission/ discharge, chest CT test, RT-PCR test and IgM/ IgG test

Patient 1 (Fig. 1), a 29-year-old man, developed a cough and a sore throat with no fever on January 28. Ground-glass opacities in chest CT and positive RT-PCR test results were obtained on February 2 and 8, respectively. Four days later (February 12), this patient developed mild pneumonia and was hospitalized for treatment (600 mg of antiviral arbidol, orally, every 12 h). From February 14 to 17, four consecutive RT-PCR test results (every day) using his throat swab specimens were all negative. With remission of pneumonia symptoms and absorption of ground-glass opacities, the patient was discharged on February 21. However, on March 5, his RT-PCR test result was tested positive again during his follow-up, and he was hospitalized again in the next day. After two negative RT-PCR test results on March 10 and 12, the patient was discharged on March 15. No evidence showed that the patient’s immune function was compromised (Table 1). Analyses of a large range of laboratory results revealed that most of tests were normal, including the Immunoglobulin G, M, A (IgG, IgM, IgA) and Complement 3, 4 (C3, C4) (Table S1). IgM and IgG were repeatedly tested using his serum samples by two different detection methods (see Methods for details) on March 7 and 8, which were all negative.

**Table 1:** Summary of Laboratory Examination Results of Two Patients

Laboratory test	Patient 1	Patient 2	Reference range
Lymphocytes, 10^9^/L	1.87	0.62	1.1–3.2
CD3+ T cells, %	81.29	53.52	58.17–84.22
CD4+ T cells, %	40.14	34.79	25.34–51.37
CD8+ T cells, %	37.66	12.49	14.23–38.95
B cells, %	13.33	24.24	4.10–18.31
NK cells, %	4.10	18.98	3.33–30.47
CD4/CD8	1.07	2.79	0.41–2.72
IL-2, pg/mL	3.93	4.04	0.10–4.10
IL-4, pg/mL	3.56	3.56	0.10–3.20
IL-6, pg/mL	4.41	681.69	0.10–2.90
IL-10, pg/mL	3.95	10.08	0.10–5.00
TNF-α, pg/mL	37.11	3.29	0.10–23.00
IFN-γ, pg/mL	3.58	3.14	0.10–18.00
IgG, au/mL	2.13	3.05	0.00–10.00
IgM, au/mL	3.21	2.67	0.00–10.00

### Case 2

Patient 2 (Fig. 1), a 58-year-old man, developed a fever and a cough on January 24 and were admitted to Wuhan Central Hospital on January 31. Multifocal ground-glass opacities were observed on chest CT images and the RT-PCR test for SARS-CoV-2 was positive on February 1. Despite anti-infection treatment and oxygen support, his symptoms worsened over the next few days, leading to severe pneumonia. On February 11, the patient was transferred to Wuhan Union Hospital for further treatment (400 mg of moxiflxacin, daily; 1000 mg of tienam, every 8 h; 200 mg of arbidol, every 8 h; 40 mg of methylprednisolone, every 12 h). The patient with glucocorticoid therapy showed reduced lymphocyte numbers and low ratios of CD3, CD4 and CD8 T cells (Table 1), suggesting his compromised immune functions. Elevated IL-6 level was related to his severe pneumonia as previously reported [4]. Moreover, 27 of 62 laboratory tests of this patient were abnormal, such as elevated levels of C-reactive protein (CRP), neutrophils and lactate dehydrogenase (LDH), and decreased levels of albumin, and hemoglobin (Table S2). On February 17 and March 11, he received two consecutive RT-PCR tests, which were positive. However, IgM and IgG in his serum samples remained undetectable on March 4, 9, and 14.

## Discussion and conclusions

Serological tests have been widely utilized in the diagnosis of COVID-19. IgM could be detected as early as 1 day post- symptom onset (PSO) and was detectable in 85% of COVID-19 confirmed patients 7 days PSO [5]. As for IgG, over 90% of COVID-19 confirmed patients produced this type of antibody 14 days after illness [5]. In this study, two patients with confirmed COVID-19 failed to produce either IgM or IgG even 40 to 50 days after their symptoms onset. Given that all of COVID-19 patients reportedly had sero-positive for IgM and IgG approximately 35 days after symptoms onset [6], the window period for antibody production in these two COVID-19 patients may be much longer, possibly up to 50 days or even longer (if they eventually produce antibodies). These finding suggests that at least a small proportion of patients may have difficulty in rapidly gaining immunity against SARS-CoV-2. The cross-reactivity among different coronaviruses may lead to false-positive results in IgM and IgG detection [6], but few false-negative outcomes were yielded, especially jointly using CLIA and ICG techniques. Thus, the fact that two or three times of negative results in the two patients using two different kits suggests that the results are unlikely to be false-negative. Analyses of the laboratory results showed that the 58-year-old patient (Case 2) had compromised immune functions, which might contribute to the negative IgM/ IgG results. However, the young patient (Case 1), who possessed normal immune functions and did not have any underlying disease, yet fails to produce IgM/ IgG 40 days after symptom onset. The reason remains unclear.

A further long-term follow-up should be carried out for these patients to determine whether they produce IgM/ IgG or persistently remain with no antibodies. Additionally, recurrence of COVID-19 was observed in the 29-year-old patient. If our finding of pneumonia recurrence in COVID-19 patients without antibodies is replicated, the management of these no-antibody patients needs to be more cautious as they may be prone to recurrence or re-infection. In summary, this work presents two typical cases of COVID-19 patients without producing IgM/ IgG and comprehensively characterizes their initial symptoms, chest CT results, medication, and laboratory test results, hoping to draw more attentions to this type of patients.

## Supplementary information

**Additional file 1: Table S1.** Laboratory Examination Results of Patient 1. **Table S2.** Laboratory Examination Results of Patient 2.

## Data Availability

The data-sets used during the current study are available from the corresponding author on reasonable request.

## Abbreviations

COVID-19: Coronavirus disease 2019
RT-PCR: Real-time polymerase chain reaction
CT: Computed tomography
IgG: Immunoglobulin G antibodies
IgM: Immunoglobulin M antibodies
ICG: Immune colloidal gold
CLIA: Chemiluminescence immunoassay
PSO: Post- symptom onset
