# Supplementary material for: COVID-19 confirmed patients with negative antibodies results
Source: BMC Infect Dis. 2020 Sep 22;20:698. doi: 10.1186/s12879-020-05419-3 (PMC7508244; doi:10.1186/s12879-020-05419-3)
Supplement: Supplementary file 1 — Additional file 1: Table S1. Laboratory Examination Results of Patient 1. Table S2. Laboratory Examination Results of Patient 2. [file 12879_2020_5419_MOESM1_ESM.doc]

**Table S1.** Laboratory Examination Results of Patient 1

| **Laboratory test** | **Patient 1** | **Reference range** | **Normal/ abnormal** |
| --- | --- | --- | --- |
| C-reactive protein, mg/L | <3.14 | <8.00 | Normal |
| Immunoglobulin G, g/L | 8.87 | 7.51-15.60 | Normal |
| Immunoglobulin A, g/L | 1.72 | 0.82-4.53 | Normal |
| Immunoglobulin M, g/L | 0.708 | 0.460-3.040 | Normal |
| C3, g/L | 0.887 | 0.790-1.520 | Normal |
| C4, g/L | 0.199 | 0.160-0.380 | Normal |
| Calcitonin, μg/L | <0.13 | <0.5 | Normal |
| Immunoglobulin E, IU/ml | 333.8 | 1-190 | Abnormal |
| Albumin/Globulin | 2.1 | 1.5-2.5 | Normal |
| Albumin | 45.8 | 35-55 | Normal |
| ALP, U/L | 76 | 40-150 | Normal |
| ALT, U/L | 27 | 5-40 | Normal |
| AST, U/L | 17 | 8-40 | Normal |
| BUN, mmol/L | 3.86 | 2.9-8.2 | Normal |
| Ca, mmol/L | 2.31 | 2.03-2.54 | Normal |
| Cl, mmol/L | 102 | 96-108 | Normal |
| Carbon dioxide, mmol/L | 24.2 | 21.0-30.0 | Normal |
| Creatinine | 76.9 | 44.0-133.0 | Normal |
| γ-GGT, U/L | 18 | 11-50 | Normal |
| Globulin | 21.4 | 20-30 | Normal |
| K, mmol/L | 4.14 | 3.5-5.2 | Normal |
| Mg, mmol/L | 0.88 | 0.70-1.1 | Normal |
| Na, mmol/L | 139.7 | 136-145 | Normal |
| P, mmol/L | 1.16 | 0.96-1.62 | Normal |
| Total bilirubin, μmol/L | 11.1 | 5.1-19.0 | Normal |
| Total protein, g/L | 67.2 | 64-83 | Normal |
| Uric acid, μmol/L | 380 | 208-428 | Normal |
| Total cholesterol, mmol/L | 3.46 | <5.2 | Normal |
| CK, U/L | 73 | 38-174 | Normal |
| HDL-C, mmol/L | 1.05 | 1.16-1.42 | Abnormal |
| LDH, U/L | 183 | 109-245 | Normal |
| Triglycerides, mmol/L | 1.63 | <1.7 | Normal |
| Anion gap, mmol/L | 14 | 8-16 | Normal |
| DBIL, μmol/L | 3.9 | 1.7-6.8 | Normal |
| Glucose, mmol/L | 4.84 | 3.90-6.10 | Normal |
| LDL-C, mmol/L | 1.96 | 2.7-3.1 | Normal |
| Osmotic pressure, mOsm/L | 296.4 | 280-310 | Normal |
| Pre-albumin, g/L | 0.328 | 0.17-0.42 | Normal |
| Total bile acid, μmol/L | 2.5 | 0-10.0 | Normal |
| HBDB, U/L | 126 | 72-182 | Normal |
| CysC, mg/L | 0.71 | 0.63-1.25 | Normal |
| LAP, U/L | 25 | 12-37 | Normal |
| 5'-NT, U/L | 6 | 0-10 | Normal |
| CKMB, U/L | 14 | 0-24 | Normal |
| Homocysteine,μmol/L | 10.5 | <15 | Normal |
| Amyloid A, mg/L | 4.6 | <10.0 | Normal |
| SdLDL, mmol/L | 1.05 | 0.234-1.378 | Normal |
| ESR, mm/h | 5 | <15 | Normal |
| MCHC, g/L | 344 | 316-354 | Normal |
| MCH, pg | 31 | 27-34 | Normal |
| MCV, fl | 89.9 | 82-100 | Normal |
| HCT, % | 41.8 | 40-50 | Normal |
| HGB, g/L | 144 | 130-175 | Normal |
| RBC, 10^12^/L | 4.65 | 4.3-5.8 | Normal |
| PDW, % | 10.9 | 15.9-17.2 | Abnormal |
| PCT, % | 0.23 | 0.1-0.28 | Normal |
| MPV, fl | 9.7 | 6.5-12.5 | Normal |
| PLT, 10^9^/L | 239 | 125-350 | Normal |
| Basophils, 10^9^/L | 0.03 | <0.06 | Normal |
| Eosinophils, 10^9^/L | 0.29 | 0.02-0.52 | Normal |
| Monocytes, 10^9^/L | 0.46 | 0.1-0.6 | Normal |
| Lymphocyte, 10^9^/L | 1.87 | 1.1-3.2 | Normal |
| Neutrophils, 10^9^/L | 3.07 | 1.8-6.3 | Normal |
| Basophils, % | 0.5 | 0-1 | Normal |
| Eosinophils, % | 5.1 | 0.4-8.0 | Normal |
| Monocytes, % | 8 | 3-10 | Normal |
| Neutrophils, % | 53.5 | 40-75 | Normal |
| WBC, 10^9^/L | 5.74 | 3.5-9.5 | Normal |
| RDW-SD, fl | 38.2 | 39.0-46.0 | Normal |
| RDW-CV, % | 11.8 | <14.5 | Normal |

**Table S2.** Laboratory Examination Results of Patient 2

| **Laboratory test** | **Patient 2** | **Reference range** | **Normal/ abnormal** |
| --- | --- | --- | --- |
| C-reactive protein, mg/L | 80.56 | <8.00 | Abnormal |
| Calcitonin, μg/L | 0.28 | <0.5 | Normal |
| Albumin/Globulin | 1 | 1.5-2.5 | Abnormal |
| Albumin | 29.6 | 35-55 | Abnormal |
| ALP, U/L | 76 | 40-150 | Normal |
| ALT, U/L | 36 | 5-40 | Normal |
| AST, U/L | 23 | 8-40 | Normal |
| BUN, mmol/L | 10.63 | 2.9-8.2 | Abnormal |
| Ca, mmol/L | 1.93 | 2.03-2.54 | Abnormal |
| Cl, mmol/L | 98.6 | 96-108 | Normal |
| Carbon dioxide, mmol/L | 37.7 | 21.0-30.0 | Abnormal |
| Creatinine | 53 | 44.0-133.0 | Normal |
| γ-GGT, U/L | 74 | 11-50 | Abnormal |
| Globulin | 30.1 | 20-30 | Abnormal |
| K, mmol/L | 3.49 | 3.5-5.2 | Normal |
| Mg, mmol/L | 0.72 | 0.70-1.1 | Normal |
| Na, mmol/L | 143.6 | 136-145 | Normal |
| P, mmol/L | 0.79 | 0.96-1.62 | Normal |
| Total bilirubin, μmol/L | 9.2 | 5.1-19.0 | Normal |
| Total protein, g/L | 59.7 | 64-83 | Abnormal |
| Uric acid, μmol/L | 230.5 | 208-428 | Normal |
| Total cholesterol, mmol/L | 3.18 | <5.2 | Normal |
| CK, U/L | 41 | 38-174 | Normal |
| HDL-C, mmol/L | 1.28 | 1.16-1.42 | Normal |
| LDH, U/L | 446 | 109-245 | Abnormal |
| Triglycerides, mmol/L | 0.99 | <1.7 | Normal |
| Anion gap, mmol/L | 4 | 8-16 | Abnormal |
| DBIL, μmol/L | 5.4 | 1.7-6.8 | Normal |
| Glucose, mmol/L | 5.11 | 3.90-6.10 | Normal |
| LDL-C, mmol/L | 2.47 | 2.7-3.1 | Normal |
| Osmotic pressure, mOsm/L | 311.8 | 280-310 | Abnormal |
| Pre-albumin, g/L | 0.28 | 0.17-0.42 | Normal |
| Total bile acid, μmol/L | 3.5 | 0-10.0 | Normal |
| HBDB, U/L | 146 | 72-182 | Normal |
| CysC, mg/L | 0.84 | 0.63-1.25 | Normal |
| CKMB, U/L | 5 | 0-24 | Normal |
| MCHC, g/L | 308 | 316-354 | Abnormal |
| MCH, pg | 30 | 27-34 | Normal |
| MCV, fl | 30 | 82-100 | Abnormal |
| HCT, % | 23.9 | 40-50 | Abnormal |
| HGB, g/L | 73 | 130-175 | Abnormal |
| RBC, 10^12^/L | 2.45 | 4.3-5.8 | Abnormal |
| PDW, % | 15.7 | 15.9-17.2 | Abnormal |
| PCT, % | 0.26 | 0.1-0.28 | Normal |
| MPV, fl | 9.5 | 6.5-12.5 | Normal |
| PLT, 10^9^/L | 273 | 125-350 | Normal |
| Basophils, 10^9^/L | 0.02 | <0.06 | Normal |
| Eosinophils, 10^9^/L | 0.03 | 0.02-0.52 | Normal |
| Monocytes, 10^9^/L | 0.98 | 0.1-0.6 | Abnormal |
| Neutrophils, 10^9^/L | 13.87 | 1.8-6.3 | Abnormal |
| Basophils, % | 0.1 | 0-1 | Normal |
| Eosinophils, % | 0.2 | 0.4-8.0 | Abnormal |
| Monocytes, % | 6.4 | 3-10 | Normal |
| Lymphocytes, % | 2.7 | 20-50 | Abnormal |
| Neutrophils, % | 90.6 | 40-75 | Abnormal |
| WBC, 10^9^/L | 15.31 | 3.5-9.5 | Abnormal |
| RDW-CV, % | 14 | <14.5 | Normal |
| BNP, pg/ml | 543.4 | <100 | Abnormal |
| Myoglobin, ng/ml | 193.9 | <146.9 | Normal |
| hs-cTn, ng/ml | 18.4 | <26.2 | Normal |
| Ferritin, ng/ml | >2000 | 21.81-274.66 | Abnormal |
